# Which person is my trainer? Spontaneous visual discrimination of human individuals by bottlenose dolphins (*Tursiops truncatus*)

**DOI:** 10.1186/s40064-015-1147-8

**Published:** 2015-07-16

**Authors:** Masaki Tomonaga, Yuka Uwano, Sato Ogura, Hyangsun Chin, Masahiro Dozaki, Toyoshi Saito

**Affiliations:** Primate Research Institute, Kyoto University, Kanrin 41-2, Inuyama, Aichi 484-8506 Japan; Port of Nagoya Public Aqualium, Minato-machi 1-3, Minato, Nagoya, Aichi 455-0033 Japan; Graduate School of Humanities, Kwansei-Gakuin University, Uegahara, Nishinomiya, Hyogo 662-8501 Japan

**Keywords:** Bottlenose dolphins, Individual recognition, Spontaneous visual discrimination, Comparative cognitive science

## Abstract

Bottlenose dolphins are known to use signature whistles to identify conspecifics auditorily. However, the way in which they recognize individuals visually is less well known. We investigated their visual recognition of familiar human individuals under the spontaneous discrimination task. In each trial, the main trainer appeared from behind a panel. In test trials, two persons (one was the main trainer) appeared from the left and right sides of the panel and moved along the poolside in opposite directions. Three of the four dolphins spontaneously followed their main trainers significantly above the level of chance. Subsequent tests, however, revealed that when the two persons wore identical clothing, the following response deteriorated. This suggests that dolphins can spontaneously discriminate human individuals using visual cues, but they do not utilize facial cues, but body area for this discrimination.

## Background

In all social-living animals, including humans, recognition of other individuals is one of the most important social-cognitive abilities (e.g., Tibbetts and Dale 2007). Each species uses various cues for individual recognition. For example, primates use mainly visual cues, such as the face (Tomonaga 1999; Tomonaga and Matsuzawa 1993; Parr et al. 1998, 2000; Dahl et al. 2013), whereas some species use scent cues (Thom and Hurst 2004). Auditory cues, especially vocalizations, also play a role in individual recognition among various species (e.g., baboons: Cheney and Seyfarth 2007; sea lions: Gwilliam et al. 2008; and crows: Kondo et al. 2010).

The bottlenose dolphin (*Tursiops truncatus*) is one of the species known to use auditory (vocal) cues for individual recognition. They emit whistles that vary individually, and it has been suggested that those whistles, called signature whistles, are used for individual recognition (Janik 2000; Janik et al. 2006; Quick and Janik 2012). However, cetaceans’ visual recognition of individuals is less well understood. Killer whales’ (*Orcinus orca*) coloration patterns differ among individuals, and certain researchers argue that killer whales may use these differences as visual cues for individual recognition (Mobley and Helweg 1990). Bottlenose dolphins also have unique visual markings, such as cookie-cutter shark bites and scars (Mikura Island Tourism Association 2010), which they may also use for visual individual recognition, although we only infer that they might identify others visually from empirical, but indirect, evidence. For example, bottlenose dolphins visually inspect marked parts of the body in front of a mirror, which has been regarded as a sign of mirror self-recognition (e.g., Reiss and Marino 2001). This suggests that they may discriminate self from others based on visual cues (but see Harley 2013 on different interpretations). Sakai et al. (2006a, b) found that Indo-Pacific bottlenose dolphins (*T. aduncus*) showed flipper-rubbing behavior with specific individuals. Furthermore, “rubber” dolphins showed lateral bias, often using the left flipper for rubbing. Sakai et al. argued that this bias was caused by left-eye dominance. These results also indirectly suggest that dolphins recognize individuals visually, but further observations are needed to reach a definitive conclusion.

To date, few experimental studies have examined visual recognition of individuals (conspecific or heterospecific) in dolphins (e.g., Thieltges et al. 2011; Nakahara et al. 2011; Tomonaga et al. 2014a, b; Murayama 2012). Nakahara et al. (2011) assessed visual recognition in bottlenose and Risso’s dolphins (*Grampus griseus*) using a preferential looking procedure; members of both species could discriminate (conspecific and/or heterospecific) other individuals on the basis of whole-body pictures. Murayama (2012) trained a bottlenose dolphin to discriminate human individuals visually, while Thieltges et al. (2011) report that dolphins attend to unfamiliar humans for a longer duration, as compared to familiar humans, in a preferential looking task. Dolphins appear to visually discriminate both conspecific and heterospecific individuals. However, to date, studies have been preliminary or have not directly focused on visual recognition of individuals. Furthermore, in some studies, explicit discrimination training with very few numbers of examples was used (e.g., Murayama 2012). It has been pointed out repeatedly that if explicit reinforcement training is introduced, animals often began to use simpler cues to solve the task, which experimenters did not expect (cf. Heyes 1993). Thus, it is still unclear whether dolphins discriminate human individuals spontaneously in their everyday life.

**Figure 1:**
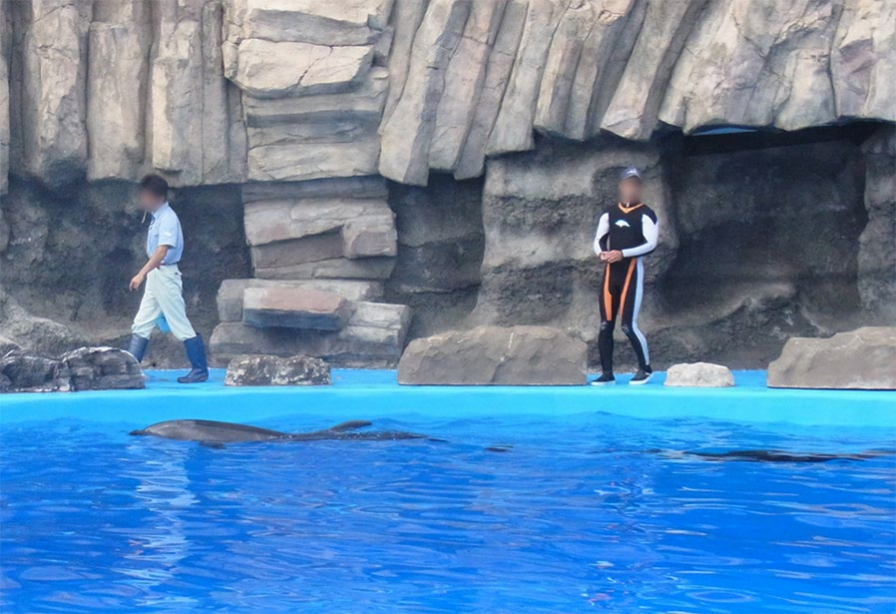
During daily, routine training at the Port of Nagoya Public Aquarium, captive bottlenose dolphins followed their main trainer. Human faces are blurred for privacy (photo courtesy of Masaki Tomonaga).

**Figure 2:**
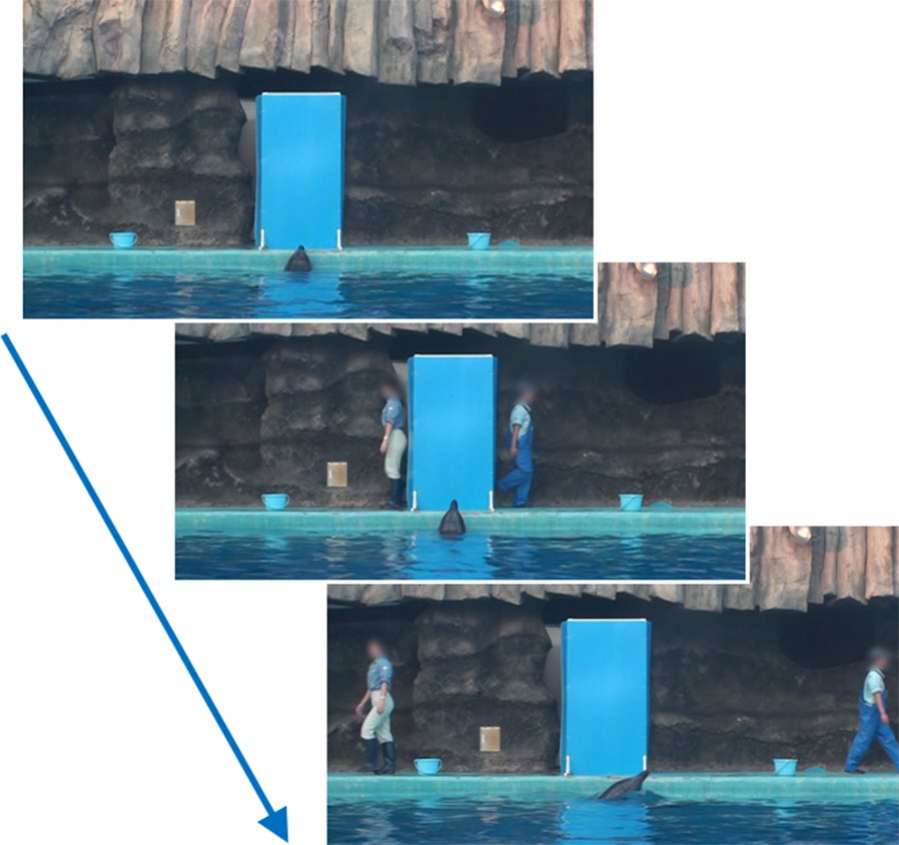
During the test trial (different-clothing condition), two persons appeared from behind the panel and walked along the poolside, in a *leftward* or *rightward* *direction*. In this trial, the dolphins’ main trainer walked in *a rightward direction*. Human faces are blurred for privacy (photos courtesy of Masaki Tomonaga).

In the present study, to test visual recognition of human individuals while avoiding the possibility of simple association learning during the course of training and testing, we introduced a “spontaneous” discrimination task in which dolphins were not differentially rewarded for following specific persons. During daily, routine training, captive bottlenose dolphins followed the human trainer frequently as he walked along the poolside (Figure 1; cf. Murayama 2012). In our paradigm, two people, one of whom was the dolphin’s main trainer, walked in opposite directions (Figure 2). Instances in which dolphins followed their own trainer were taken as evidence of visual recognition of human individuals.

## Results

Four adult male bottlenose dolphins were the participants in the present experiment, which was conducted in the Port of Nagoya Public Aquarium, Nagoya City, Aichi, Japan. An opaque, blue panel was positioned on one side of the pool (Figure 2).

During the “different-clothing” condition, the two people (i.e., the main trainer and the “dummy” person) wore different clothes; they both appeared from behind the panel, walked to the left or right along the poolside, and met at the opposite side of the pool (Figure 2). Dolphins followed one of the two persons. When dolphins crossed to the opposite side of the pool, the main trainer performed a gestural command. The dolphin was rewarded only after successfully performing the action corresponding to the gestural sign, irrespective of the following response. Therefore, following responses were not explicitly and differentially reinforced. Each dolphin partook in 24 test sessions following eight training sessions.

**Figure 3:**
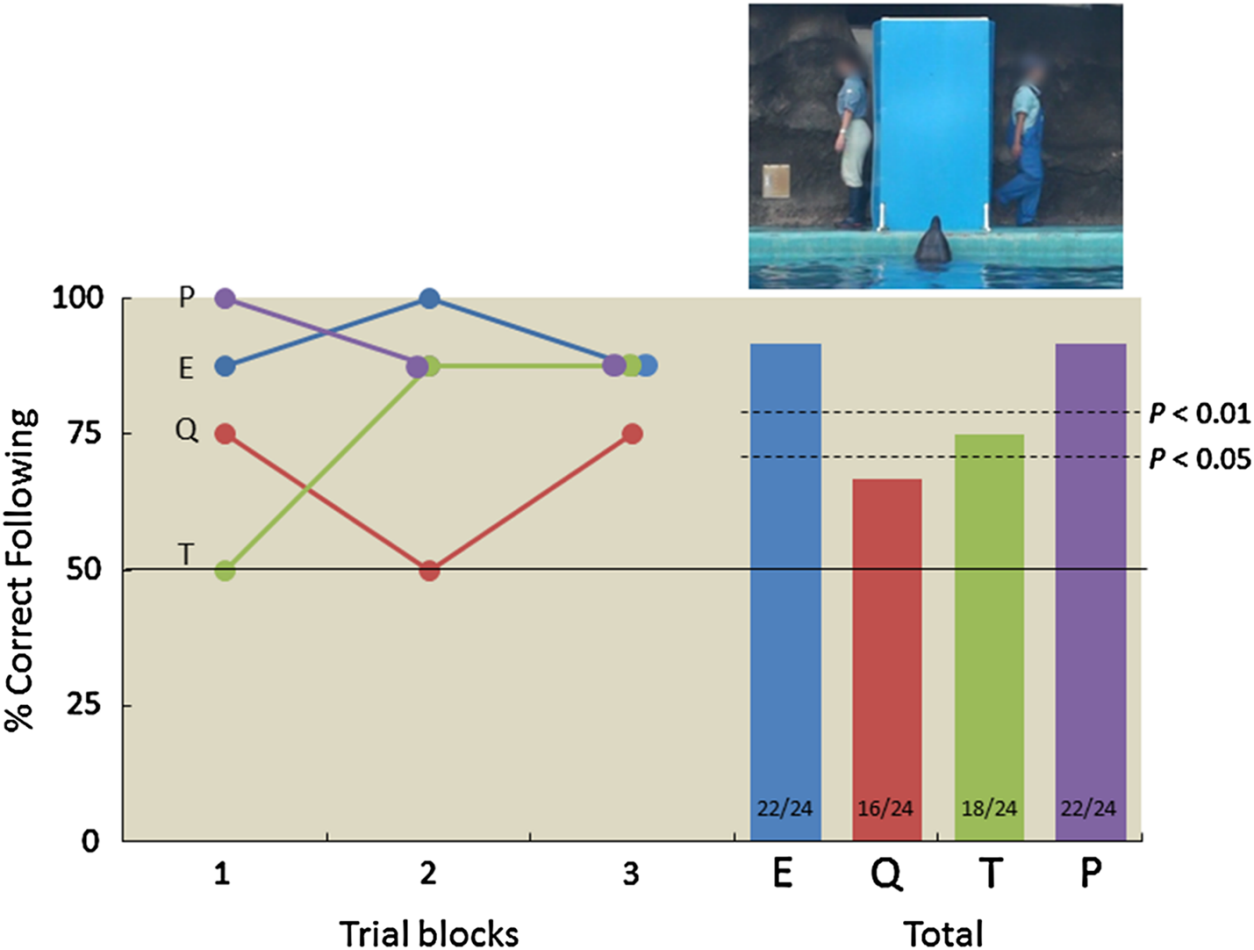
Proportion of correct following responses for each dolphin during the different-clothing condition. *Left panel* change in performance across eight-trial blocks. *Right panel* the proportion of correct responses of each dolphin averaged across sessions. *Numbers* at the bottom of each *bar* indicate the ratio between correct following responses/total trials. *Dashed lines* indicate *P* < 0.05 and *P* < 0.01 significance levels. *E* Eagle, *Q* Quick, *T* Tino, *P* Peace. Human faces are blurred for privacy (photo courtesy of Masaki Tomonaga).

The right panel of Figure 3 provides the mean percentages of correct following responses, for each dolphin, across all different-clothing test sessions. Three of the four dolphins followed the main trainer significantly more frequently than the dummy person [Eagle, *p* < 0.001; Quick, *p* = 0.076; Tino, *p* = 0.011; and Peace, *p* < 0.001 (binomial tests)]. Although the sample size was small, they followed their main trainer significantly more frequently than would be expected by chance [one-sample *t* test (vs. 50%), *t*(3) = 5.00, *p* = 0.0154, *d* = 2.50]. The left panel of Figure 3 describes change in performance across eight-trial blocks. Tino’s performance gradually improved, but not significantly. During the test trials, Quick exhibited a strong position bias; he was significantly more likely to swim to the left side (21/24 trials, *p* < 0.001), and swam to the left on every error trial (8/8, *p* = 0.004). Therefore, Quick completed eight additional test sessions following the four preliminary training sessions in which the persons did not hide behind the panel, but stayed in front of the panel. Consequently, the dolphin was able to track the main trainer visually (see “Methods” section). He committed no errors during the test trials in these preliminary training sessions. During the eight additional test sessions, he followed the main trainer on every occasion (8/8, *p* = 0.004).

**Figure 4:**
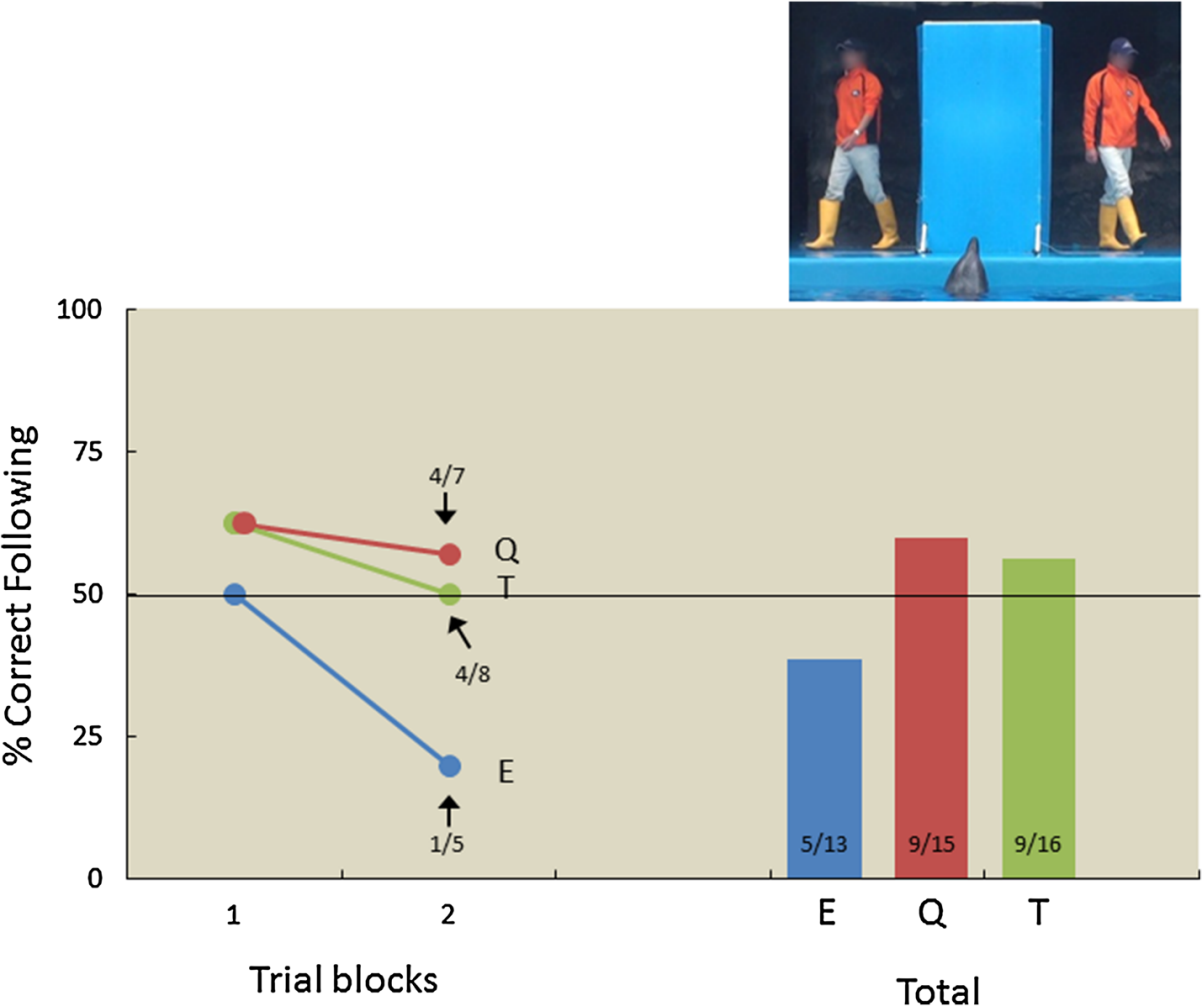
Proportion of correct following responses for each dolphin during the same-clothing condition. *Left panel* change in performance across eight-trial blocks (the second block did not consist of eight trials; therefore, the correct following responses/total trials ratio is presented near to the 1 data points). *Right panel* proportion of correct responses averaged across sessions. *Numbers* at the bottom of each *bar* indicate the ratio between correct following responses/total trials. *E* Eagle, *Q* Quick, *T* Tino. Human faces are blurred for privacy (photo courtesy of Masaki Tomonaga).

To further examine the visual cues most critical to visual discrimination, we conducted additional tests within the same-clothing condition, in which two people of the same sex, and of similar height, wore identical clothing (Figure 4). The right panel of Figure 4 denotes the proportion of correct following responses for each dolphin averaged across test sessions, while the left panel describes change in performance as a function of trial blocks. Three dolphins participated in this condition; there was no significant difference in the likelihood of dolphins following their main trainer or the dummy person (Eagle, *p* = 0.867; Quick, *p* = 0.304; Tino, *p* = 0.402). All three dolphins exhibited a strong right-sided bias (Eagle, 13/13, *p* < 0.001; Quick, 14/15, *p* < 0.001; Tino, 13/16, *p* = 0.011).

## Discussion

Although not explicitly and differentially rewarded, three of our four dolphins spontaneously followed their main trainer when the main trainer and dummy person wore different clothes. Furthermore, following reprisal of the preliminary training session, to control for position bias, the fourth dolphin also performed accurate following responses. These results suggest that dolphins discriminate human individuals visually during everyday life. However, when the main trainer and dummy person wore identical clothes, following accuracy was reduced. During this condition, trainers’ faces represented the only explicit visual cues. For numerous mammalian species, the face is the most important cue for individual recognition (Tibbetts and Dale 2007) of both conspecific and heterospecific organisms. Domestic animals, such as dogs and horses, can discriminate human faces (Adachi et al. 2007; Stone 2010). However, our data indicate that dolphins do not utilize facial cues for human recognition in this setting (cf. Murayama 2012). This is consistent with our previous study, in which trainers’ faces were masked but dolphins nevertheless readily and accurately performed actions corresponding to gestural signs (Tomonaga et al. 2010). The head region (i.e., attentional state) of the trainer did not play a critical role for our dolphins (but see Tschudin et al. 2001; Pack and Herman 2004, 2007).

The other possible cues used by the dolphins were those produced by human motion. The captive dolphins became very sensitive to human movements because they were trained to follow gestural signs. Herman et al. (1990) clearly demonstrated that the motion information produced by the point-light display is sufficient to elicit the appropriate response to the sign. Motion also provides unique individual auditory cues, such as the sounds of footsteps. Experimentally, dogs could discriminate their owner from strangers based on only the sounds of footsteps (Fujita et al. 2010). However, our dolphins likely did not use these cues in the present experiments, because if they did, their discrimination performance would not have deteriorated in the same-clothing test.

Why did our dolphins fail to utilize visual cues from facial regions during the recognition of human individuals? One possibility is that this non-use of facial (head) cues is generalized from conspecific discrimination, during which dolphins do not attend to facial cues. Cetaceans lack the facial expressions (cf. Kuczaj et al. 2013) that characterize other mammalian species, particularly primates, suggesting that the facial region is relatively unimportant during their social communications. Therefore, when dolphins do recognize conspecifics visually, it is likely that they are using other parts of the body as cues. As described previously, individual differences in coloration patterns or visual markings on the body may serve as cues (Mobley and Helweg 1990). Our data support this possibility; the dolphins appeared to discriminate between humans based on differences in bodily regions, as they probably also do when identifying conspecifics.

If this hypothesis is correct, a further issue remains to be addressed: how do dolphins match human body parts (including the face) to their own bodies? It is clear that the body structure of dolphins and humans is completely different, both anatomically and visually. Therefore, matching, for example, the rostrum to the mouth, pectoral fins to the arms, and caudal fins to the legs appears problematic. However, evidence from motor imitation studies suggests that dolphins can match body parts in this manner (Herman 2002, 2012). Bottlenose dolphins will “imitate” the movements of a human model; when the model raised his leg, the dolphins raised their caudal fins, and when the human extended his arms, dolphins extended their pectoral fins. Therefore, in the present experiment, it is plausible that our dolphins recognized the correspondence between the human head and body and their own anatomy.

## Conclusion

In conclusion, this study is the first to demonstrate systematically that captive bottlenose dolphins spontaneously discriminate human individuals visually using bodily rather than facial cues. These data also suggest that dolphins are likely to discriminate conspecifics using visual information provided by body parts. Of course, this conclusion does not exclude the possibility that dolphins can visually discriminate between the faces of humans, conspecifics, and so on if they are explicitly and differentially trained (cf. Murayama 2012). Further experimental studies on the visual recognition of conspecifics are required in the future (Tomonaga et al. 2014a).

## Methods

### Participants

Four adult male bottlenose dolphins (*Tursiops truncatus*), Eagle, Quick, Tino, and Peace, participated in the present experiments. They lived as a group in the Port of Nagoya Public Aquarium in Nagoya City, Aichi, Japan. They usually received four 15-min sessions of husbandry, performance, and cognitive training per day by the human trainers (Tomonaga et al. 2010, 2014a, b).

### Experimental setting

**Figure 5:**
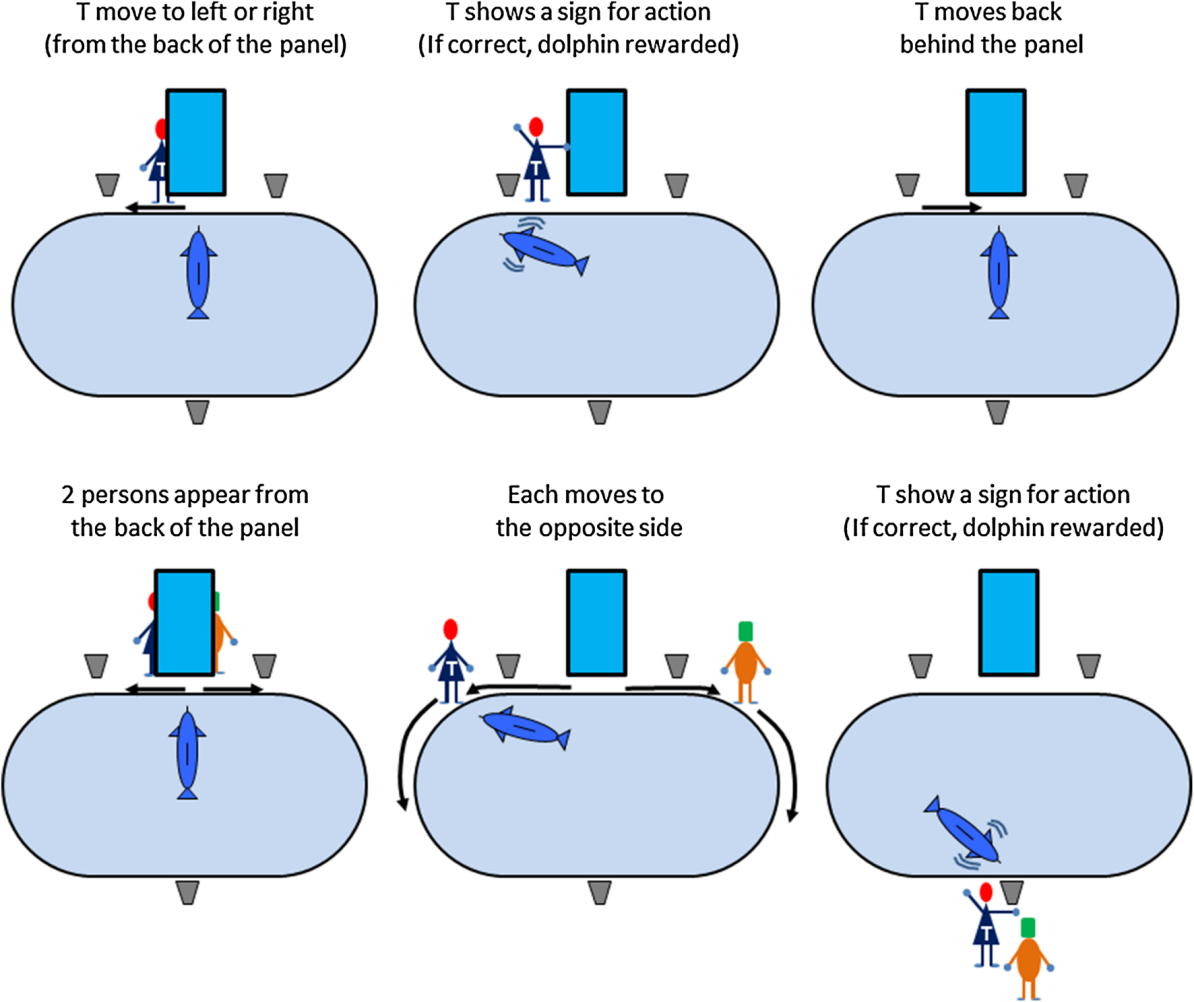
Schematic diagram of the baseline (*upper*) and test (*lower*) trials in the different-clothing test. *T* main trainer.

Experiments were conducted in a pool (elliptical shape, 34 × 18 m and 9 m in depth) adjacent to their home pool (Figure 5). At the one side of the pool, a blue opaque panel (PVC, 90 cm wide, 180 cm high) was set (Figure 2). Identical buckets with rewards (pieces of fish) were placed on the floor 1 m to the left- and right-side edges of the panel. Another bucket was placed at the opposite side of the pool.

### Procedure

#### Preliminary training

Each dolphin was initially given preliminary training sessions, which consisted of five trials. The first four were baseline trials, and the last one was the test trial. Before the onset of the trial, the main trainer stood in front of the panel. Once the dolphin stayed in front of the panel, the trainer moved to left or right and showed a gestural sign for action (e.g., jump, flipper-shaking) by the dolphin. If the dolphin performed the corresponding action correctly, a whistle was sounded, and a food reward was given. After the reward, the trainer made the dolphin move to the front of the panel and stay there, and the trainer also stayed in front of the panel. In the baseline trials, only the main trainer appeared. In the test trial, two persons (of the same sex) stood in front of the panel simultaneously. One of them was the main trainer, and the other was a dummy person (but familiar to the dolphin). Each person then walked to the left or right along the pool side, and they met at the opposite side of the pool. In daily routine training, when trainers walked along the pool side, the dolphins always followed them. Thus, in this test setting, the dolphin also followed one of the persons. When the dolphin came to the opposite side where the third bucket was located, the main trainer performed a gestural command to the dolphin. The dolphin was rewarded only when it successfully performed the action corresponding to the gestural sign, irrespective of the following response. Thus, following responses were not explicitly and differentially reinforced. Each dolphin was given eight preliminary training sessions.

During preliminary training sessions completed before each test session, all dolphins followed the main trainers on every trial.

#### Different-clothing test

After completing the preliminary training, each dolphin was given 24 test sessions (i.e., 24 test trials). Figure 5 shows the flow of the baseline and test trials. The difference between the preliminary training and the subsequent test trials was that in the latter, the trainer always hid behind the panel. In the different-clothing test trials, the two people wore different clothes to each other, and the main trainer also changed clothes randomly from session to session (Figure 2). The side from which the main trainer appeared (which also set the direction in which the trainer then walked) was counterbalanced and randomized. During the preliminary training and testing sessions, each dolphin–trainer pairing was fixed. Dummy persons were changed from session to session.

#### Same-clothing test

One year after the different-clothing test, we conducted an additional test in which the two persons were of the same sex and of similar height, and they wore identical clothing (Figure 4). A different trainer from the first test was assigned to each dolphin. Before the test sessions, the dolphins were given 12 preliminary training sessions in which the two persons stood in front of the panel; however, due to the limitations imposed by exhibition schedules, only three dolphins (Eagle, Quick, and Tino) participated in this test; they were given 13, 15, and 16 sessions, respectively.

Throughout the experiments, dolphins responded accurately to the gestural commands given by the trainer (97.9% accuracy, averaged across dolphins).
